# BYHWD Alleviates Inflammatory Response by NIK-Mediated Repression of the Noncanonical NF-κB Pathway During ICH Recovery

**DOI:** 10.3389/fphar.2021.632407

**Published:** 2021-05-07

**Authors:** Wei Xiao, Zehui He, Weikang Luo, Dandan Feng, Yang Wang, Tao Tang, Ali Yang, Jiekun Luo

**Affiliations:** ^1^ Department of Integrated Traditional Chinese and Western Medicine, Institute of Integrative Medicine, Xiangya Hospital, Central South University, Changsha, China; ^2^ Department of Orthopedics, Movement System Injury and Repair Research Center, Xiangya Hospital, Central South University, Changsha, China; ^3^ Department of Neurology, Henan Provincial People’s Hospital, People’s Hospital of Zhengzhou University, Zhengzhou, China

**Keywords:** buyang huanwu decoction, the noncanonical NF-κB pathway, inflammation, NF-κB inducing kinase, intracerebral hemorrhage

## Abstract

Intracerebral hemorrhage (ICH) is a life-threatening type of stroke that lacks effective treatments. The inflammatory response following ICH is a vital response that affects brain repair and organism recovery. The nuclear factor κB (NF-κB) signaling pathway is considered one of the most important inflammatory response pathways and one of its response pathways, the noncanonical NF-κB signaling pathway, is known to be associated with persistent effect and chronic inflammation. NF-κB–inducing kinase (NIK) *via* the noncanonical NF-κB signaling plays a key role in controlling inflammation. Here, we investigated potential effects of the traditional Chinese medicine formula Buyang Huanwu Decoction (BYHWD) on inflammatory response in a rat model of ICH recovery by inhibiting the NIK-mediated the noncanonical NF-κB signaling pathway. In the first part, rats were randomly divided into three groups: the sham group, the ICH group, and the BYHWD group. ICH was induced in rats by injecting collagenase (type VII) into the right globus pallidus of rats' brain. For the BYHWD group, rats were administered BYHWD (4.36 g/kg) once a day by intragastric administration until they were sacrificed. Neurological function was evaluated in rats by a modified neurological severity score (mNSS), the corner turn test, and the foot-fault test. The cerebral edema showed the degree of inflammatory response by sacrificed brain water content. Western blot and real-time quantitative reverse transcription PCR tested the activity of inflammatory response and noncanonical NF-κB signaling. In the second part, siRNA treatment and assessment of inflammation level as well as alterations in the noncanonical NF-κB signaling were performed to determine whether the effect of BYHWD on inflammatory response was mediated by suppression of NIK *via* the noncanonical NF-κB signaling pathway. We show that BYHWD treated rats exhibited: (i) better health conditions and better neural functional recovery; (ii) decreased inflammatory cytokine and the edema; (iii) reduced expression of NIK, a key protein in unregulated the noncanonical NF-κB signaling pathways; (iv) when compared with pretreated rats with NIK targeting (NIK siRNAs), showed the same effect of inhibiting the pathway and decreased inflammatory cytokine. BYHWD can attenuate the inflammatory response during ICH recovery in rats by inhibiting the NIK-mediated noncanonical NF-κB signaling pathway.

## Introduction

Intracerebral hemorrhage (ICH) is part of the most devastating types of cerebrovascular disease threatening human health. It suffers approximately 2 million people globally each year with a high mortality rate (Cordonnier et al., 2018). Moreover, most of the survivors must endure varying degrees and kinds of life-long disabilities that result from both primary and secondary brain injury. Although the understanding of ICH-induced primary and secondary brain damage has improved (Wang, 2010; Lan et al., 2017), ICH therapy for long-term recovery is still eager to explore.

Since therapies focus on the primary injury of ICH have had limited success, which has led to a center of attention on secondary injury mechanisms (Feigin et al., 2003). Inflammatory cytokines are key participants in secondary injury after ICH. The nuclear factor–κB (NF-κB) signaling pathway plays an important role in the processes of inflammation, immunity, cancer, and neural plasticity (Mattson and Meffert, 2006; O'Sullivan et al., 2010; Perkins, 2007). The NF-κB family of transcription factors is activated by canonical and noncanonical signaling pathways, which differ in both signaling components and biological functions. The NF-κB signaling pathway has been identified as an important signaling pathway in secondary brain injury, after ICH (Sun, 2017; Zhu et al., 2019). The noncanonical NF-κB signaling pathway is characterized by selectively slow, persistent, and specific, strictly relies on phosphorylation of IKK1 by NIK and then induce p100 processing and nuclear translocation to play a role (Sun, 2011). Most studies have demonstrated that the noncanonical NF-κB signaling pathway regulates important physiological functions such as lymphoid organogenesis, B-cell survival and maturation, dendritic cell activation, and bone metabolism (Dejardin, 2006). Besides, dysregulation of this pathway is associated with different diseases, such as lymphoid malignancies, immunodeficiency, abnormal hematopoiesis, metabolic disorders, and vascular injury (Cildir et al., 2016). Therefore, the regulation of this pathway is important to explore potential ways to modulate inflammation by targeting key factors and then to ameliorate inflammatory response and promote neurological function recovery after ICH. Besides, the NF-κB pathways have been studied as therapeutic targets in a variety of acute and chronic brain diseases and the role remains to be elucidated, especially concerning the noncanonical NF-κB pathway. Therefore, a better understanding of this mechanism regulating unregulated NF-κB activation has important therapeutic value.

Traditional Chinese medicine (TCM) as one of the important treatments for stroke has been used for thousands of years. Buyang Huanwu decoction (BYHWD), a classical TCM formula, is the most frequently used for treating stroke (40.32%) by far (Hung et al., 2015). Evidence shows BYHWD substantial neuroprotective and function-improving effects in animal models of focal cerebral ischemia and desirable clinical efficacy for both hemorrhagic and ischemic stroke (Guo et al., 2015; Luo et al., 2017; Pan et al., 2017). What’s more, BYHWD improves long-term motor dysfunctions, swallowing difficulties, cognitive disorders, and dysarthria, which are tough to be effective by Western medicine (Zhang et al., 2016a; Xu et al., 2017). However, the multifaceted regulatory mechanisms of BYHWD acting on ICH have not been fully clarified.

Here, we expand upon previous studies and explore the effect of the noncanonical NF-κB pathway in the recovery period of ICH. Our findings may provide a novel perspective to illustrate the molecular mechanisms of BYHWD treatment for long-term neurological function recovery after ICH.

## Materials and Methods

### Animals

Adult male Sprague–Dawley rats weighing 180–220 g were obtained from the Experimental Animal Center of Central South University (CSU), Changsha, China. All rats were maintained under specific pathogen-free (SPF) conditions with a 12-hour light/dark cycle, a controlled temperature (25°C), a relative humidity (45–55%), and ad libitum had access to water and food pellets throughout the study. All procedures were conducted following the guidelines and approved by the Institutional Animal Care and Use Committee of CSU (No: 2018sydw0159).

### Collagenase Model

The collagenase-induced ICH rat model was an important way for studying ICH and had been used by various researchers. All experimental rats were deeply anesthetized intraperitoneally with 3% pentobarbital sodium (50 mg/kg) and placed in a stereotaxic frame (Stoelting Co., Chicago, IL, United States). After disinfection and incision, a hole was drilled in the skull and collagenase type VII (Sigma-Aldrich, United States, 0.5 U in 2.5 μL of 0.9% sterile saline) was injected slowly (over 2 min) *via* microliter syringe into the right globus pallidus, according to the following coordinates relative to bregma: 1.4 mm posterior, 3.2 mm lateral, and 6 mm ventral to the cortical surface. The needle stayed for an additional 10 min to prevent reflux then slowly removed. Sham group rats underwent the same procedure, except that the syringe contained 2.5 μL of 0.9% saline solution without collagenase. Due to the practical operation of the experiment, NIK siRNAs group rats underwent the same procedure but syringed into the left globus pallidus.

### Preparation of BYHWD

BYHWD was prepared and subjected to quality control as previously described (Cui et al., 2015). *Astragalus mongholicus* Bunge [Fabaceae; Astragali Radix], *Angelica sinensis (Oliv.) Diels* [Apiaceae; Angelica Sinensis Radix], *Paeonia lactiflora* Pall*.* [Paeoniaceae; Paeonia Radix Rubra], *Ligusticum Chuanxiong* Hort [Apiaceae; Chuanxiong Rhizoma], C*arthamus tinctorius* L. [Asteraceae; Carthami Flos], *Prunus persica* (L.) Batsch [Rosaceae; Persicae Semen], and *Pheretima aspergillum* (E. Perrier) [Megascolecidae; Pheretima] at a ratio of 60:6:4.5:3:3:3:3 (dry weight) were used (Table 1). All botanical drugs were available from the Chinese Medicinal Pharmacy of Xiangya Hospital, Central South University (Changsha, China). Briefly, all botanical drugs were immersed in distilled water for 1-h, and boiled for 30 min at 100°C. Finally, the powder (yield: 11.9%) was dissolved in distilled water at a concentration of 0.13 g/ml for intragastric administration.

**TABLE 1:** Components of the Buyang Huanwu Decoction.

Scientific name	Chinese name	English name	Family	Medicinal part	Batch number
*Astragalus mongholicus* Bunge	Huang Qi	Astragali Radix	Fabaceae	Root	19070913
*Angelica sinensis* (Oliv.) Diels	Dang Gui	Angelica Sinensis Radix	Apiaceae	Rhizome	20190704
*Paeonia lactiflora* Pall.	Chi Shao	Paeonia Radix Rubra	Paeoniaceae	Root	19062607
*Ligusticum Chuanxiong* Hort	Chuan Xiong	Chuanxiong Rhizoma	Apiaceae	Root	1906290
*Carthamus tinctorius* L.	Hong Hua	Carthami Flos	Asteraceae	Flower	19050804
*Prunus persica* (L.) Batsch	Tao Ren	Persicae Semen	Rosaceae	Seed	19061010
*Pheretima aspergillum* (E. Perrier)	Di Long	Pheretima	Megascolecidae	Whole animal	19051802

Above listed botanical drugs were combined in a 60:6:4.5:3:3:3:3 ratio (dry weight). All plant names or species were validated using http://www.worldfloraonline.org/or https://mpns.science.kew.org/mpns-portal/searchName or https://www.drugfuture.com/standard/.

### Qualitative Analysis of BYHWD

We purchased the standard reference materials of amygdalin, hydroxysafflor yellow A, calycosin, and digoxin from Yuanye Bio-Technology Co., Ltd. (Shanghai, China). Digoxin is not the endogenous compound of BYHWD and plasma, and it does not obviously interfere with the retention times of all three analytes. Qualitative analysis was carried out using an LC-MS system (Shimadzu 8050, Kyoto, Japan) in negative ion mode. The plasma samples were added with digoxin, vortex mixed for 10 s, and then vortex mixed for 1 min and centrifuged for 15 min (13,000 rpm, 4°C) after being added 800 μL acetonitrile. The obtained supernatants were dried in a nitrogen dryer, diluted with 10% acetonitrile–water, repeated the extraction above, and injected into the LC-MS for analysis (Supplementary Figure S1).

### Experimental Design

There were two parts to the entire experiment. In the first part, rats were randomly divided into three groups: the sham group, the ICH group, and the BYHWD group. For the BYHWD group, rats were administered by gastrogavage with BYHWD once daily for the duration of the experimental observations after ICH modeling. According to our previous study (Cui et al., 2015), we chose 4.36 g/kg as the dosage of BYHWD in the present study. The distilled water with equal volumes was administered to the sham and ICH groups. Rats were sacrificed on days 3, 7, and 14, post-ICH after neural tests. In the second part, intact rats first randomly received a microinjection of NIK targeting (siNIK) or non-targeting siRNA (siControl) into the right lateral ventricles at concentrations of 10 mM. The siNIK group (NIK siRNAs + ICH) received NIK siRNAs (NIK siRNAs, Cyagen Biosciences, aCSF, 10 μL, i.c.v.) to inhibit the noncanonical NF-κB. The control group (siRNAs control + Sham and siRNAs control + ICH) received scramble siRNAs (siRNAs control, Cyagen Biosciences, aCSF, 10 μL, i.c.v.). Both were injected immediately with a cannula implantation system (RWD Life Science) once per day for 5 days. ICH surgery was conducted after 5 days of recovery. ICH induction provided a detailed description in the “collagenase model” previously. The BYHWD was administered to the ICH BYHWD group as before, and distilled water with equal volumes was administered to the siRNAs control + Sham, the siRNAs control + ICH, and the NIK siRNAs + ICH. Rats were sacrificed at days 7 and 14 after ICH, respectively.

### Behavioral Tests

The modified neurological severity score (mNSS), the corner turn test, and the foot-fault test were used to assess the neurological injury at various time points after ICH by two observers who blinded to the group assignments independently and their scores were averaged. These tests were detailedly performed in our previous studies (Narantuya et al., 2010; Cecatto et al., 2014; Krafft et al., 2014).

### mNSS

The modified neurological severity score (mNSS) examined motor ability, walking ability, sensory ability, balance, reflexes, and the presence of abnormal movements. And a higher score meant a more severe injury (normal score: 0; maximal deficit score: 18).

### Corner Turn Test

Rats would proceed into a corner with an angle of 30°degrees. To exit the corner, individual rats could turn either left or right, and the direction taken was then recorded. This was repeated 10 times per animal, with at least 30 s between trials. The percentage of right turns was calculated. The percentage of right turn in normal rats was about 50%, while the injured ones would have a higher percentage.

### Foot-Fault Test

Rats were tested for placement dysfunction of forelimbs with the modified foot-fault test. Rats were set on an elevated grid surface (85.8 × 2.5 cm^2^, with grids of different sizes) and placed their paws on the wire while moving along the grid. With each weight-bearing step, the paw may fall or slip between the wire, which was recorded as a foot fault. The total number of steps (movement of each forelimb) that the rats used to cross the grid was counted, and the total number of foot faults for the left forelimb was recorded. Data are presented as the percentage of foot faults per the total number of steps (normal score: 0%; maximal deficit score: 100%).

### Sample Preparation

Rats were deeply anesthetized with 3% pentobarbital sodium (50 mg/kg, i.p.). For molecular biology experiments, including Western blot and quantitative real-time polymerase chain reaction (RT-qPCR), rats were perfused with 0.9% saline, and brain samples around the hematoma were collected in ice-cold saline. They were immediately stored in liquid nitrogen to ensure brain tissue staying fresh then stored at −80°C until analysis.

### Brain Water Content

Rats were sacrificed at days 3, 7, and 14 after ICH induction, and the intact brain tissues were removed immediately. Brain tissues were divided into two hemispheres along the midline and the ipsilateral hemisphere divided into three parts by stainless steel brain matrices. The hematoma area brain tissue was weighed using an electronic analytical balance. After drying in an oven at 95°C for 72 h, until the sample weights were nearly consistent, the dry weight was obtained and calculated as follows: water content of brain tissues (%) = (wet weight–dry weight)/(wet weight) × 100%.

### Western Blot Analysis

Collected fresh samples were homogenized in RIPA lysis buffer with a protease inhibitor. The homogenate was centrifuged at 12,000 rpm for 30 min at 4 °C. The supernatants were collected immediately. Protein samples were separated by SDS-PAGE gels and transferred onto PVDF membranes (Millipore, United States). The membranes were blocked with 5% BSA for 2 h at room temperature and then probed with primary antibodies overnight with gentle shaking at 4°C. After three washes in PBS-T, the membranes were subsequently incubated with horseradish peroxidase–conjugated anti-mouse IgG (1:5000, Proteintech, United States) or anti-rabbit IgG (1:6000, Proteintech, United States) secondary antibodies for 2 h at room temperature. Membranes were washed again, and the protein bands were visualized using enhanced chemiluminescence (ECL). The exposed films were scanned and analyzed by Quantity One analysis software.

The primary antibodies used were the following: rabbit anti-NIK (1:200, Abcam, United Kingdom); rabbit anti-IKKα (1:1000, Cell Signaling Technology, United States); rabbit anti-NF-κB p100/52 (1:1000, Cell Signaling Technology, United States); mouse anti-β-actin (1:5000, proteintech, United States); rabbit anti-Histone H3 (1:1000, Cell Signaling Technology, United States); rabbit anti-TNF-α (1 ug/ml, Abcam, United Kingdom); and rabbit anti-IL-β (0.2 ug/ml, Abcam, United Kingdom).

### RT-qPCR

Total RNA was extracted with a TRIzol reagent (Invitrogen, Carlsbad, CA, United States) following the manufacturer’s instructions. Amplification was performed using SYBR Green All-in-one TM qPCR Mix (GeneCopoeia) on a ViiA^TM^7 RT-qPCR system (Applied Biosystems). The following thermocycling protocol was used: 95°C for 10 min, 40 cycles of 15 s at 95°C, 50 s at 60°C, and melting was done at 60°C. The primers for IL-1β, TNF-α, and β-actin were designed with Premier 5.0 software for rats. The sequences of the primers are shown in Table 2. Melting curves of all the samples were generated as controls for specificity. The expression data were normalized to the expression of β-actin with the 2^−ΔΔCt^ method.

**TABLE 2:** The RT-qPCR sequences of primers.

Gene	Primer	Sequence (5′ to 3′)	PCR Product length (bp)
NIK	Forward	TCA​TCG​CGG​GGT​CAC​AGC​AGT​ACA	156
	Reverse	ACT​TCG​ACC​TCC​TCT​TCC​TAC​GTT	
TNF-α	Forward	AAA​GCA​TGA​TCC​GAG​ATG​TGG​AA	142
	Reverse	AGT​AGA​CAG​AAG​AGC​GTG​GTG​GC	
IL-1β	Forward	ACT​TGG​GCT​GTC​CAG​ATG​AG	114
	Reverse	GTA​GCT​GCC​ACA​GCT​TCT​CC	
β-actin	Forward	ACA​TCC​GTA​AAG​ACC​TCT​ATG​CC	223
	Reverse	TAC​TCC​TGC​TTG​CTG​ATC​CAC	

### *In Vivo* Lateral Ventricles Injection

NIK targeting (NIK siRNAs) or non-targeting siRNA (siRNAs control) was injected into the right lateral ventricles of rats according to the following coordinates relative to bregma: 1 mm posterior, 1.5 mm lateral, and 4 mm ventral to the cortical surface at concentrations of 10 mM. 10 µL of either the NIK siRNAs or siRNAs control were injected into the lateral ventricle at 1 μL/min. The siNIK group (NIK siRNAs + ICH) received NIK siRNAs (NIK siRNAs, Cyagen Biosciences, aCSF, 10 μL, i.c.v.) to inhibit the noncanonical NF-κB. The control group (siRNAs control + sham and siRNAs control + ICH) received scramble siRNAs (siRNAs control, Cyagen Biosciences, aCSF, 10 μL, i.c.v.). Both were injected immediately with a cannula implantation system (RWD Life Science) once per day for 5 days. The inhibitory effect of NIK siRNA is shown in Supplementary Figure S2. The rat Map3k14 shRNA design and siRNAs control target sequences (NIK siRNAs) were: CCT​TGG​AAA​GGA​GAA​TAT​AAA and scramble-shRNA-PGK (siRNA control): CCT​AAG​GTT​AAG​TCG​CCC​TCG.

### Statistical Analysis

Results were presented as the mean ± SEM. One-way analysis of variance (ANOVA) was used for comparing differences. A Levene test was used to test the variance congruence of the data. Post hoc tests for multiple comparisons, using the LSD or the Bonferroni test if the variance is equal, or Tamhane's test if the variance is not equal. A *p*-value of <0.05 by SPSS 24 software was considered as statistically significant.

## Results

### BYHWD Attenuated Neurological Recovery Impairment After ICH

**FIGURE 1:**
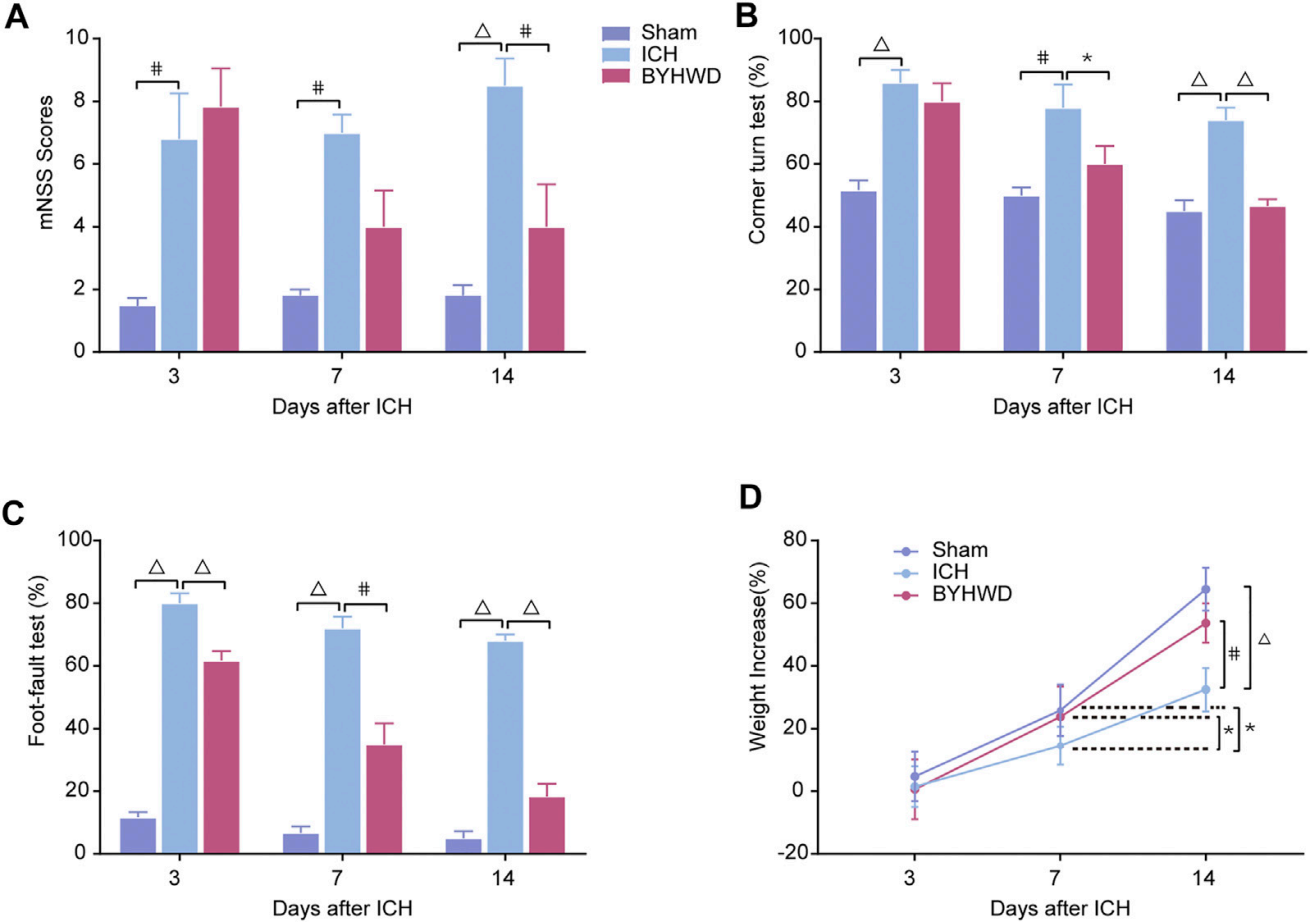
BYHWD attenuated neurological recovery impairment after ICH. On day 3, the mNSS **(A)**, the corner turn test **(B)**, the foot-fault test **(C)**, and the weight increased rate **(D)** of ICH and BYHWD group were significantly different with the sham group, which indicated the successful ICH models. On day 14, rats in the BYHWD group showed a significantly lower mNSS **(A)** as well as better performance in corner turn **(B)** and foot-fault tests **(C)** and the weight increased rate **(D)** than the ICH group, which suggested the therapeutic effects of BYHWD for functional recovery after ICH. Data were presented as mean ± SEM (*n* = 6 each group); *p < 0.05, #*p* < 0.01, ^Δ^ *P* < 0.001. mNSS, modified neurological severity score; weight increased, the difference between weight values of days 3, 7, and 14.

To define the effects of BYHWD in neurological behavioral impairment after ICH, we recorded the body weight and performed behavioral testing 1 h before sacrifice at each time point. Recovery of neurological function after ICH was achieved by BYHWD treatment; all results are shown in Figure 1. All rats were observed to be healthy and with normal body functions before modeling. Day 3 after ICH, there was little weight gain in collagenase-induced rats (ICH and BYHWD groups), while the sham group grew normally (Figure 1D). Meanwhile, the ICH and BYHWD group rats had severe neurological behavioral impairment compared to the sham group, which also showed the success of ICH modeling. Substantially higher scores of mNSS showed from the ICH (*p* < 0.01) and BYHWD groups, respectively (Figure 1A). Similarly, the percentages of the ICH and BYHWD groups were notably higher than the sham in the corner turn test (Figure 1B) and the foot-fault test (Figure 1C). Day 7 after ICH, the test scores of ICH rats were still higher than sham in mNSS (*p* < 0.001), the corner turn test (*p* < 0.01), and the foot-fault test (*p* < 0.001), while the scores of BYHWD rats were gradually decreasing. Day 14 after ICH, the test scores of ICH rats had remained unchanged as before and were still higher than sham in mNSS (*p* < 0.001, Figure 1A), the corner turn test (*p* < 0.01, Figure 1B), and the foot-fault test (*p* < 0.001, Figure 1C). However, after 14-day treatment with BYHWD, the neurological function impairment of the BYHWD group was ameliorated at varying degrees in mNSS (Figure 1A), the corner turn test (Figure 1B), and the foot-fault test (Figure 1C). Additionally, for weight change (Figure 1D), the ICH group grow slowly and the weight of the BYHWD group gained about over 20%.

### Decreased Expression and Secretion of Inflammatory Cytokines After BYHWD Treatment of ICH

**FIGURE 2:**
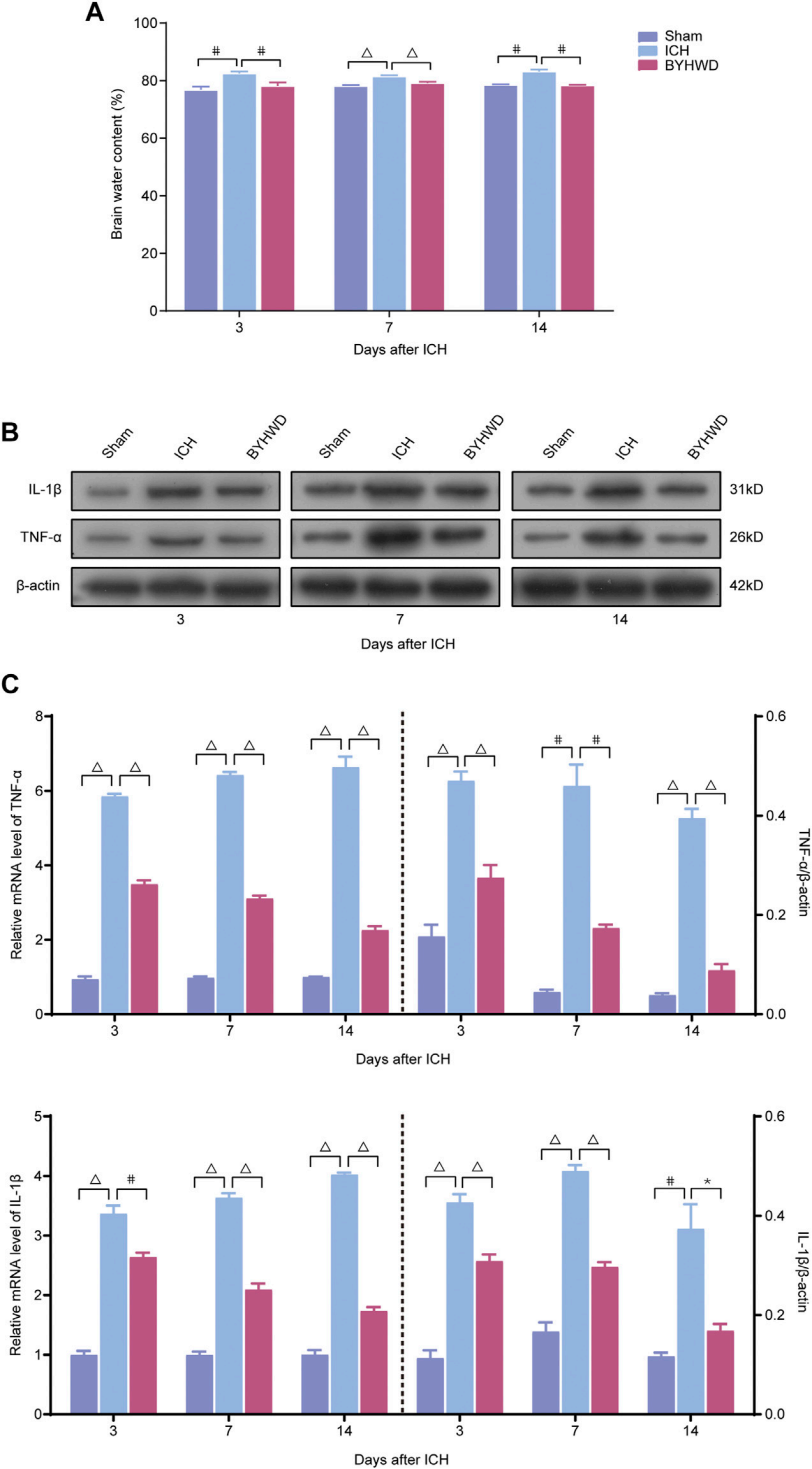
BYHWD reduced the degree of cerebral edema and downregulated the inflammatory factors following ICH. Recorded brain water content **(A)** on days 3, 7, and 14 after ICH. A representative immunoblot showed the effects of ICH and BYHWD on the protein levels of inflammatory factors TNF-α and IL-1β **(B)** at the ipsilateral tissue. The left side of the dotted line shows the relative mRNA levels of TNF-α and IL-1β **(C)**, respectively. The right side of the dotted line shows the protein levels of TNF-α and IL-1β **(C)**, respectively. Levels of TNF-α and IL-1β mRNAs were dramatically decreased in the BYHWD-treated group after ICH. Western blot analysis also showed that the expression levels of TNF-α and IL-1β were significantly downregulated after ICH. BYHWD suppressed inflammatory response. Relative TNF-α and IL-1β levels were calculated based on densitometry analysis. The mean TNF-α and IL-1β level of the sham group was normalized to 1.0. Data were displayed as mean ± SEM (*n* = 6 each group); *p < 0.05, #*p* < 0.01, ^Δ^ *P *< 0.001 deemed as significant difference.

As shown in Figure 2, to explore the effects of BYHWD treatment on ICH-induced inflammation in brain tissues, we examined brain edema using a wet/dry method on days 3, 7, and 14, evaluating the impact of BYHWD treatment on ICH-induced brain edema with its dynamic variations (Figure 2A). In the ipsilateral hematoma area of these three groups, the ICH group showed significant increases in water content compared with the Sham group, while BYHWD treatment considerably alleviated the brain water content on day 14 after ICH. Inflammatory cytokine activation contributed to the evolution of secondary degeneration after ICH, both mRNA and protein levels of TNF-α and IL-1β, which were the most important indicators of inflammation response and were detected by RT-qPCR and Western blot analysis on days 3, 7, and 14 after ICH (Figures 2B,C). All the observation time points, the results showed that these two indicators were increased in the ICH group compared with the sham group. However, the BYHWD group significantly decreased both expression levels compared with the ICH group as treatment time progressed. Treatment with BYHWD remarkably decreased the IL-1β and TNF-α secretion after ICH, compared with the ICH group. Above all, inflammatory cytokine could be well inhibited by BYHWD. The results demonstrated that the use of BYHWD may contribute to the control of ICH-induced inflammation.

### NIK was Highly Expressed in ICH Rats’ Brain Tissues and BYHWD Inhibited the Activity of the Noncanonical NF-κB Signaling in Brain Tissues After ICH

**FIGURE 3:**
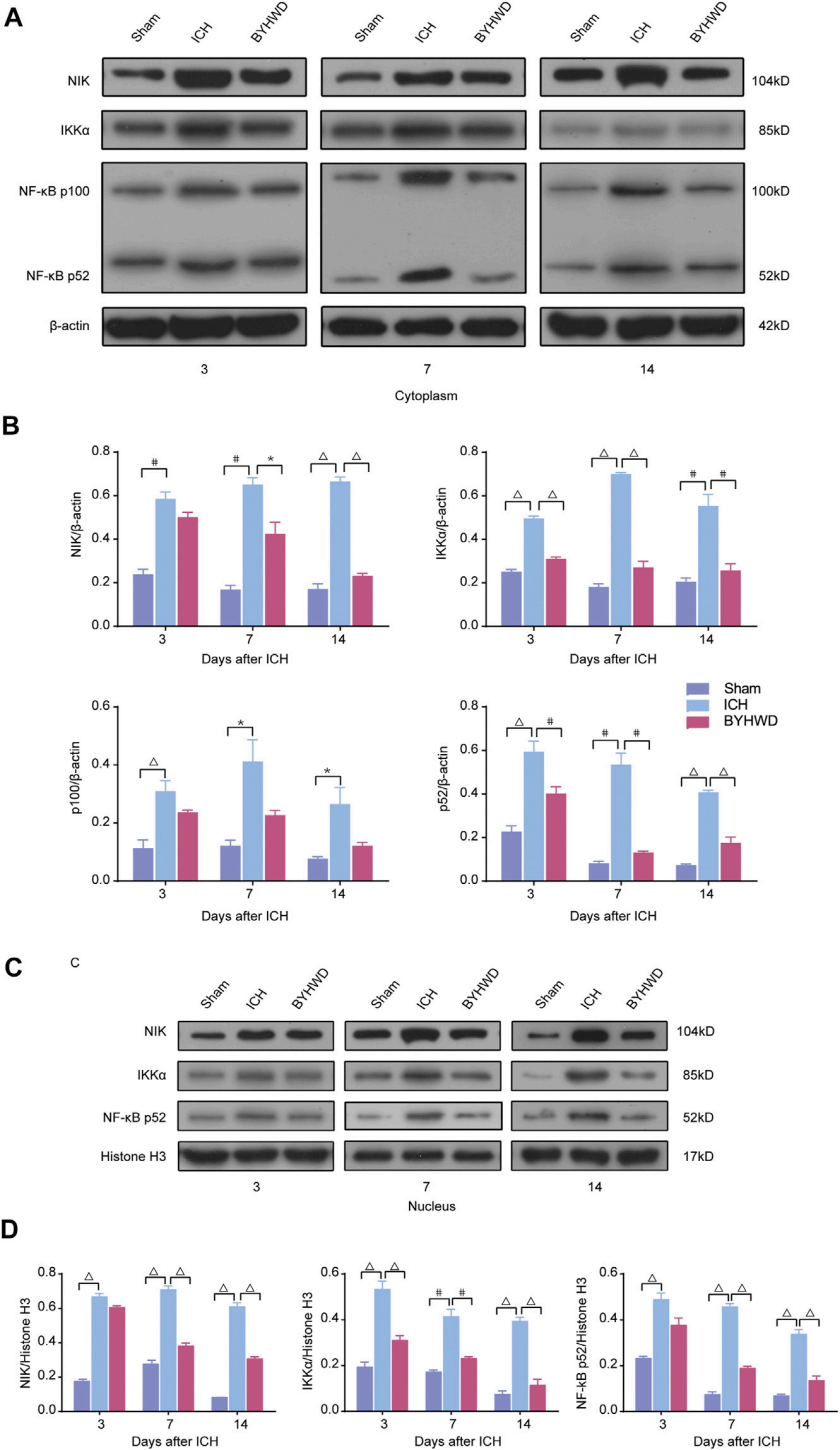
NIK was highly expressed in ICH rats’ brain tissues and BYHWD inhibited the activity of the noncanonical NF-κB signaling after ICH. Activation of the noncanonical NF-κB pathway on days 3, 7, and 14 after ICH in cytoplasm and nucleus. A representative immunoblot showed the effects of ICH and BYHWD on the protein levels of key factors, NIK, Ikkα, NF-κB p100, and NF-κB p52, in the noncanonical NF-κB pathway in cytoplasm **(A)** and the nucleus **(C)** at the ipsilateral tissue. Relative expression of NIK, Ikkα, NF-κB p100, and NF-κB p52 protein in cytoplasm **(B)** and NIK, Ikkα, and NF-κB p52 protein **(D)** in the nucleus. Data are the mean ± SEM (*n* = 6 each group); *p < 0.05, #*p* < 0.01, ^Δ^ *P* < 0.001 deemed as significant difference.

We investigated the specific indicators of the noncanonical NF-κB pathway, including NIK, Ikkα, NF-κB p100, and NF-κB p52, dynamic changes of protein expression after ICH using Western blot (Figures 3A–D).

The results showed that several specific indicators of the noncanonical NF-κB pathway, including NIK, Ikkα, NF-κB p100, and NF-κB p52, were increased in brain tissue after ICH. In the cytoplasm, day 3 after modeling, the expression levels of NIK, IKKα, NF-κB p100, and NF-κB p52 were markedly increased in the ICH group compared with the sham group. And these indicators were relatively decreased in the BYHWD treatment group compared with the ICH group. After long-term treatment, NIK, Ikkα, NF-κB p100, and NF-κB p52 in the ICH group were still in high expression on days 7 and 14 after ICH, but the BYHWD group significantly improved. In the nucleus, NF-κB p100 was not detected because it was turned into p52 before entering the nucleus. The results of NIK, Ikkα, and NF-κB p52 were consistent with that of the cytoplasm. Day 3 after modeling, compared with the sham group, the expression of NIK, IKKα, and NF-κB p52 was remarkably increased in the ICH group. The expression of these three indicators was reduced in the BYHWD group compared with the ICH group, and the decrease in IKKα turned to be statistically different. On days 7 and 14, the results were similar to those in the cytoplasm. These findings suggested that specific indicators of the noncanonical NF-κB pathway were highly expressed in ICH rats’ brain tissues and the BYHWD treatment can inhibit NIK, the key promoters of noncanonical NF-κB pathways, in brain tissues after ICH.

### BYHWD Promoted Recovery by NIK-Mediated Repression of the Noncanonical NF-κB Pathway

**FIGURE 4:**
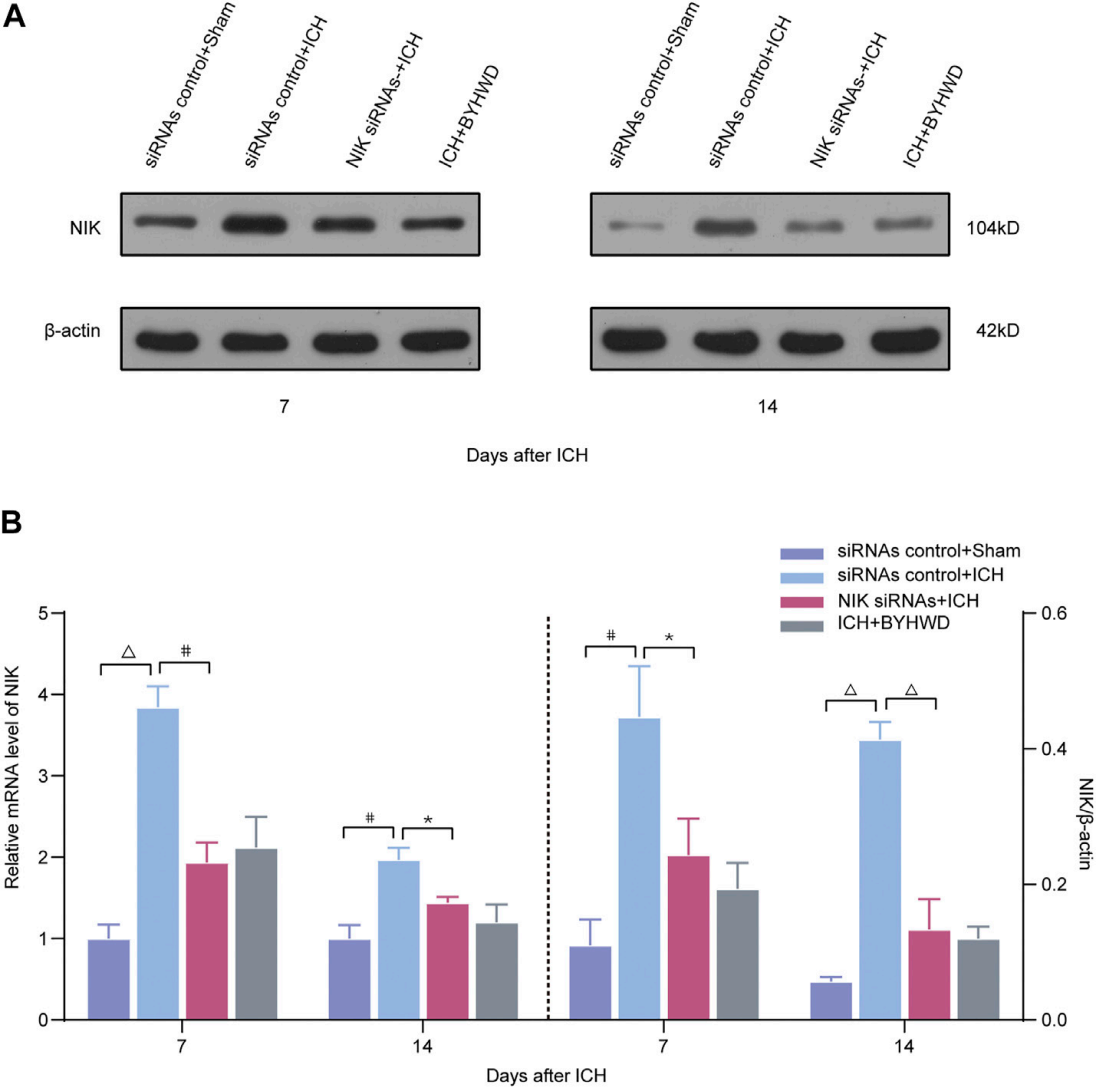
BYHWD suppresses the expression of NIK and regulates the noncanonical NF-κB pathway by targeting NIK protein *via* transfected with NIK siRNAs screening. Relative expressions of the key protein NIK were measured on days 7 and 14 transfected with NIK siRNAs or siRNAs control after ICH. **(A)** Representative immunoblot showed the effects of siRNAs control + Sham, siRNAs control + ICH, NIK siRNAs + ICH, and ICH + BYHWD on the protein levels of NIK at the ipsilateral injury area. The left side of the dotted line shows the relative mRNA levels of NIK **(B)**. The right side of the dotted line shows the protein levels of NIK. Levels of NIK mRNAs were dramatically decreased in the NIK siRNAs + ICH and ICH + BYHWD group after ICH. Western blot analysis showed that the expression levels of NIK were significantly downregulated after ICH. Effect of BYHWD treatment is consistent with the effect of blocking NIK transfected with NIK siRNAs. BYHWD suppressed the key protein. Relative NIK levels were calculated based on densitometry analysis. The mean NIK level of the sham group was normalized to 1.0. Data are the mean ± SEM (n = 3 each group); *p < 0.05, #*p* < 0.01, ^Δ^ *P *< 0.001 deemed as significant difference.

**FIGURE 5:**
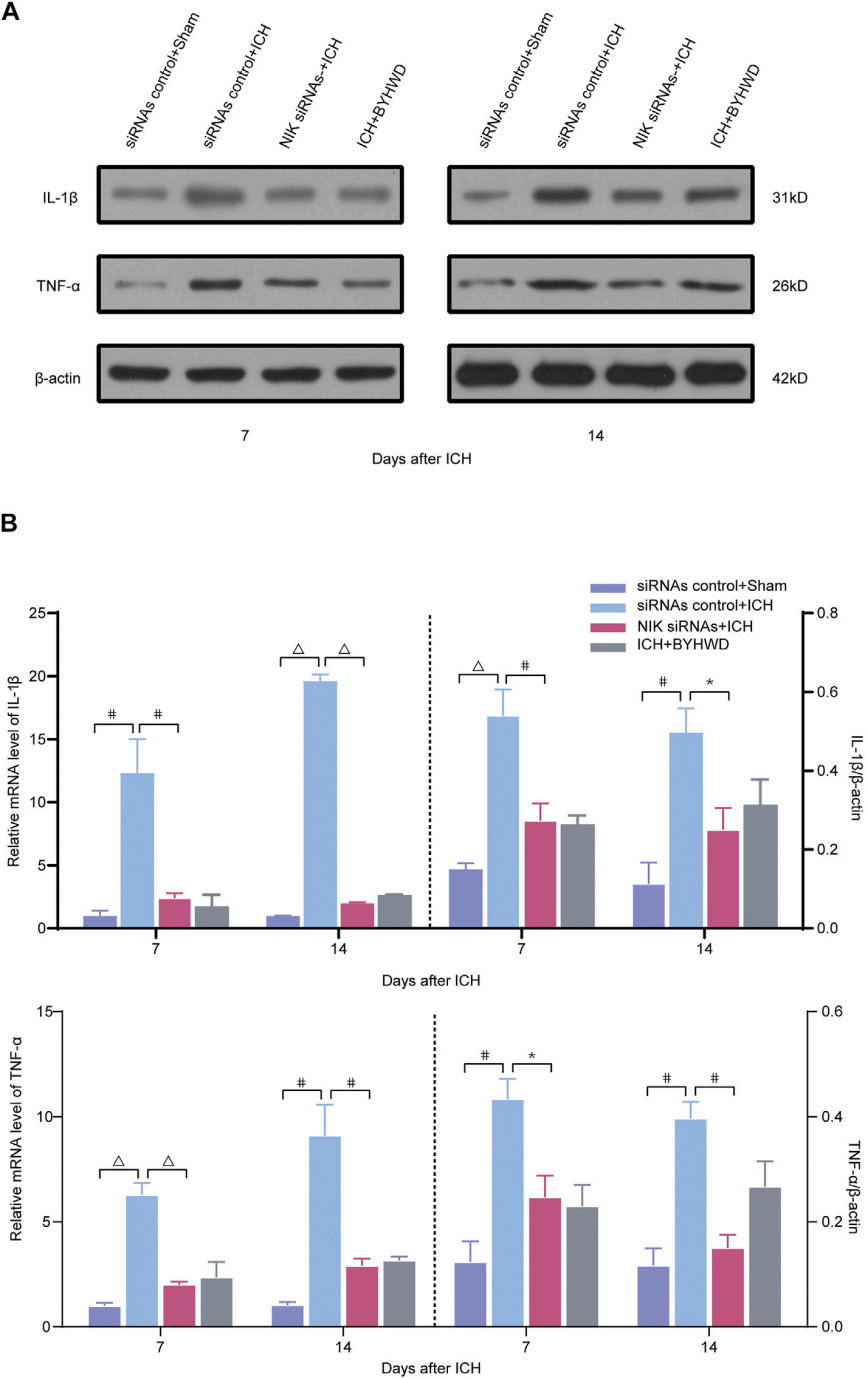
Downregulated the inflammatory factors TNF-α and IL-1β following ICH *via* transfected with NIK siRNAs screening. Relative expressions of TNF-α and IL-1β were measured on days 7 and 14 transfected with NIK siRNAs or siRNAs control after ICH. A representative immunoblot showed the effects of siRNAs control + Sham, siRNAs control + ICH, NIK siRNAs + ICH, and ICH + BYHWD on the protein levels of inflammatory factors TNF-α and IL-1β **(A)** at the ipsilateral injury area. The left side of the dotted line shows the relative mRNA levels of TNF-α and IL-1β **(B)**, respectively. The right side of the dotted line shows the protein levels of TNF-α and IL-1β **(B)**, respectively. Levels of TNF-α and IL-1β mRNAs were dramatically decreased in the NIK siRNAs + ICH and the ICH + BYHWD group after ICH. Western blot analysis showed that the expression levels of TNF-α and IL-1β were significantly downregulated after ICH. Effect of BYHWD treatment is consistent with the effect of blocking NIK transfected with NIK siRNAs. BYHWD suppressed inflammatory response. Relative TNF-α and IL-1β levels were calculated based on densitometry analysis. The mean TNF-α and IL-1β level of the sham group was normalized to 1.0. Data are the mean ± SEM (*n* = 3 each group); *p < 0.05, #*p* < 0.01, ^Δ^ *P *< 0.001 deemed as significant difference.

Following the determination that NIK is highly expressed in brain tissue after ICH, we further investigated whether targeting of NIK in ICH alone could suppress the inflammatory responses in the ICH model. To answer this, we pretreated rats with either NIK targeting (NIK siRNAs) or non-targeting siRNA (siRNAs control) before ICH surgery. Then using RT-qPCR and the Western blot analysis measured the mRNA and the protein level of NIK (Figure 4) and the inflammation indicators (Figure 5) in brain tissues on days 7 and 14 after ICH.

Compared with the siRNAs control + sham group, the relative mRNA level of NIK in each ICH group was increased at different degree (Figure 4). The results showed that the relative mRNA and protein expression of NIK in the NIK siRNAs + ICH group was downregulated than that in the siRNAs control + ICH, which represented the success of pretreatment. Compared with the NIK siRNAs + ICH group, the ICH + BYHWD group showed no statistical difference in the relative mRNA expression of NIK levels and protein levels of NIK on days 7 and 14 after ICH. But mRNA and protein levels of NIK both in the NIK siRNAs + ICH group and the ICH + BYHWD group were much lower than those in the siRNAs control + ICH group showed on days 7 and 14 after ICH. This suggests that the activation of key regulators of noncanonical NF-κB pathway was inhibited in siRNA-pretreated rats after ICH, while NIK was also inhibited by BYHWD treatment.

The results showed that inflammatory factors were notably in the brain tissue after ICH. The relative mRNA and protein expression of TNF-α/IL-1β in the NIK siRNAs group was significantly decreased than those in the control siRNA group after ICH of both observation time (Figure 5). These observations indicated that after downregulating NIK, the key regulator of the noncanonical pathway, the activation of the noncanonical pathway was inhibited. However, experiments of first part have shown that BYHWD can effectively inhibit NIK.

Relative mRNA and protein levels of inflammatory indicators were comparable between the NIK siRNAs + ICH group and the ICH + BYHWD group on days 7 and 14 after ICH, suggesting that BYHWD alleviates the inflammatory response after the recovery of ICH and may be involved in NIK-mediated repression of the noncanonical NF-κB pathway.

## Discussion

In the present study, we reported that BYHWD treatment reduced inflammation after ICH by inhibiting the NIK-mediated regulation of the noncanonical NF-κB pathway and then attenuated neurological impairment. Compared with the ICH rats, rats treated with BYHWD reduced the extent of brain edema after ICH, inflammatory factors, and inhibited the noncanonical NF-κB pathway by regulating NIK; in contrast, these effects were absent in the ICH nontreated group as well as in the siRNA control group. Finally, ICH rats treated with BYHWD showed significantly enhanced recovery and repair of neurological deficits in several classical behavioral tests.

After ICH, in addition to the primary injuries, secondary injuries were caused by pathologic responses to the hematoma with persistent effects on prognosis and recovery. The secondary injuries, including inflammation, oxidative stress, excitotoxicity, and cytotoxicity, trigger different pathophysiological changes that lead to local trauma and neurological and organismal functional impairments (Wang and Tsirka, 2005; Yang et al., 2006; Wang and Dore, 2007; Aronowski and Zhao, 2011; Wu et al., 2011; Zhou et al., 2014; Duan et al., 2016; Jiang et al., 2016; Lan et al., 2017). Inflammation, as an important factor in secondary injury, is a complicated process that is mediated mainly by cellular and molecular components. Cellular components include leukocytes, macrophages, astrocytes, T-cells, and microglia, whereas molecular components include prostaglandins, chemokines, cytokines, extracellular proteases, and ROS. Numerous studies have shown that these changes lead to inflammatory reactions, including inflammatory cell recruitment and activation, as well as inflammatory media release, which contributes to the progression of brain injury and repair (Wang and Tsirka, 2005; Wang and Dore, 2007; Wang, 2010; Mracsko and Veltkamp, 2014). NF-κB is one of the key regulators of the body’s multi-modulation signal transduction pathway in the inflammatory and neuronal apoptosis during postinjury neurogenesis (Morishita et al., 2014); activation can induce macrophage migration factors, neurotrophic factors, extracellular matrix release increase inflammatory fine cytokine expression levels, and increase the peripheral nerve inflammatory response (Chou et al., 2011). Mostly, NF-κB is a transcription factor that plays a key role in inflammatory processes (Teng et al., 2009; Xiong et al., 2016). Thus, control of the NF-κB pathway response and modulation of inflammatory factors may provide a new direction for treatment. Since the canonical NF-κB pathway occurs early after injury and responses rapidly and short-lived, whereas the noncanonical NF-κB pathway is a process in which the response continues to act. To control inflammation, almost researches focused on the canonical NF-κB pathway and few did researches on the noncanonical NF-κB pathways relatively. But the noncanonical NF-κB pathways activate in a tightly regulated way (Shih et al., 2011; Sun, 2011; Zhang et al., 2014). So, the noncanonical NF-κB pathway responses with limited stimuli and persistent feature theoretically provide a more possible therapeutic intervention, potentially limiting bad effects and targeting much severer or persistent injury. Therefore, we consider that the noncanonical NF-κB pathways play an important role during the postinjury recovery period. The noncanonical NF-κB pathway predominantly targets activation of the p52/RelB NF-κB complex by NIK. Inducers of the noncanonical NF-κB pathway discovered so far are thought to be associated with NIK signaling activation (Claudio et al., 2002; Coope et al., 2002; Dejardin et al., 2002; Kayagaki et al., 2002; Novack et al., 2003). Studies to date have shown that NIK plays a key regulatory role either deficiency and overactivity related to many diseases, such as diverse malignancies (Nadiminty et al., 2007; Gonzalez-Murillo et al., 2015), immunodeficiency (Shinkura et al., 1999; Willmann et al., 2014; Shen et al., 2017), autoimmunity (Huang et al., 2018; Liu et al., 2018), organ injuries (Ruiz-Andres et al., 2016; Ortiz et al., 2017) (Jiang et al., 2015; Liu et al., 2017; Shen et al., 2017; Xiong et al., 2018), abnormal glucose metabolism (Sheng et al., 2012; Malle et al., 2015), sarcopenia and osteopenia (Taniguchi et al., 2014; Fry et al., 2016), vascular injury (Maracle et al., 2017; Maracle et al., 2018), and so on. So, it is important to tighten control of the NIK. In our study, we detected that NIK was highly expressed in the brain tissue after ICH. The noncanonical NF-κB pathways actively activate in the injured region and associate with local and systemic inflammation, which may affect progression of ICH. From our research, BYHWD might downregulate ICH-induced NIK by inhibiting the inflammatory response. NIK continues the responses and affects the long-term recovery of ICH. Therefore, targeting NIK in the recovery phase of ICH by BYHWD can hold therapeutic potential to promote recovery. Various studies have been explored because of the complex process of ICH and the NF-κB pathway (Hayden and Ghosh, 2008). In inactivated cells, NIK levels are kept low by TRAF3, which promotes constitutive proteasomal degradation of NIK by virtue of the E3–ubiquitin ligase complex consisting of TRAF2, TRAF3, and cIAP1/2. Upon receptor stimulation, NIK is stabilized by cIAP-mediated degradation of TRAF3, which then activates and also promotes the binding of IKKα to its substrate p100, which is ubiquitinated and processed into p52 after phosphorylation (Sun, 2011; Verhelst et al., 2015). BYHWD may interfere with the formation of TRAF2, TRAF3, and CIAP1/2 and thereby increases the levels of NIK protein. However, in the process of our study, we have only preliminarily explored the effect of BYHWD on the noncanonical NF-κB pathway and focus on whether it would have an effect on the key regulators of the noncanonical NF-κB pathway. So, we may further explore its internal sophisticated mechanism of action and whether there are regulatory effects on upstream and downstream in the future. At the same time, MAP kinase–regulated pathways may act to promote survival or death, depending on the cellular context in which they are activated. From previous studies, it appears that MAP kinases are activated to some extent after ICH as a response to external stimuli (Skaper et al., 2001; Ohnishi et al., 2007; Chen et al., 2016). Furthermore, BYHWD, as a compound, is inherently multi-targeted and its mechanisms can involve in many pathways and proteins. Given the complexity of the pathophysiological response to disease and the complex and diverse processes of the inflammatory response, the mechanisms by which BYHWD acts in the treatment of ICH will be investigated further.

Previously, investigations have shown that BYHWD alleviates inflammation in many diseases in rats, such as cerebral ischemia, stroke, ischemic heart disease, Alzheimer's disease, and spinal cord injury (Iadecola and Alexander, 2001; Liu et al., 2011; Kim et al., 2020; Ryu et al., 2020). In ICH, BYHWD plays a therapeutic role through different aspects (Chen et al., 2008; Zhou et al., 2008; Cui et al., 2015; Cui et al., 2018; Kang et al., 2019; Li et al., 2019). Studies have shown that BYHWD can modulate the inflammatory response, mediate apoptosis, decrease ROS generation (Jin et al., 2013; Shen et al., 2016), promote neovascularization, improve cerebral circulation, and enhance brain tissue repair (Liu et al., 2015; Zhang et al., 2016b). Researchers focused on the NF-κB pathway with BYHWD, which was mostly limited to the canonical pathway and was dedicated to reducing the inflammatory effects of the acute phase of injury. But in fact, in the long term, it is more important for people with ICH to recover body function and improve quality of life during the recovery period. BYHWD as a compound preparation has not been systematically studied to elucidate the role of specific active ingredients in the treatment of ICH, but studies have reported the effect of some of the components of BYHWD on the inflammatory response. *Astragalus* Radix and *Angelicae sinensis* Radix are the two main components of BYHWD and have been shown to attenuate inflammation (Xu et al., 2015; Wang et al., 2016). Moreover, the hydroalcoholic extract of Radix *Astragalus* significantly reduced the activation of microglia and astrocytes (Di Cesare Mannelli et al., 2017). Nonetheless, the exact mechanism is still unclear. Thus, for the time being, we can only speculate that it is the major component of BYHWD that regulate NIK. And it is worth investigating which botanical drugs or components are targeting NIK, thereby reducing inflammation responses and promoting recovery after ICH, as well as which cells play a role in neural recovery.

## Conclusion

**FIGURE 6:**
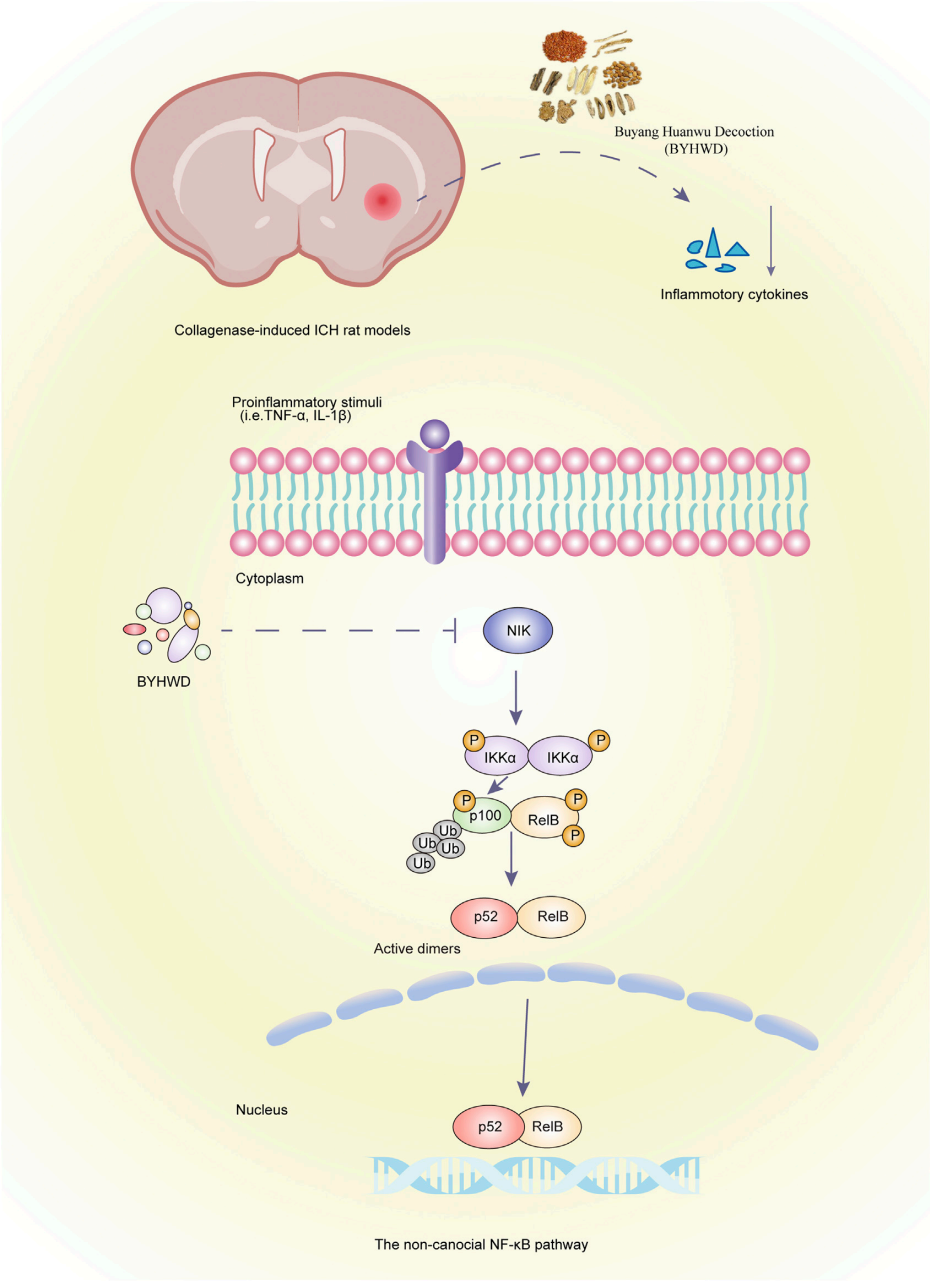
Graphic abstract. Schematic of BYHWD attenuate the inflammatory response by inhibiting the noncanonical NF-κB pathway *via* downregulation of NIK at the recovery phase of ICH in a rat model.

Taken together, our findings suggest that NIK, a key factor in the noncanonical NF-κB signaling pathway, plays an important role in the inflammatory response in ICH, and the mechanism of BYHWD for treating ICH may involve downregulation of NIK to attenuate the inflammatory response and promote recovery (Figure 6).

## Data Availability

The original contributions presented in the study are included in the article/Supplementary Material, further inquiries can be directed to the corresponding authors.
