# Supplementary material for: BYHWD Alleviates Inflammatory Response by NIK-Mediated Repression of the Noncanonical NF-κB Pathway During ICH Recovery
Source: Front Pharmacol. 2021 May 7;12:632407. doi: 10.3389/fphar.2021.632407 (PMC8138445; doi:10.3389/fphar.2021.632407)
Supplement: Supplementary file 1 [file DataSheet1.docx]

**
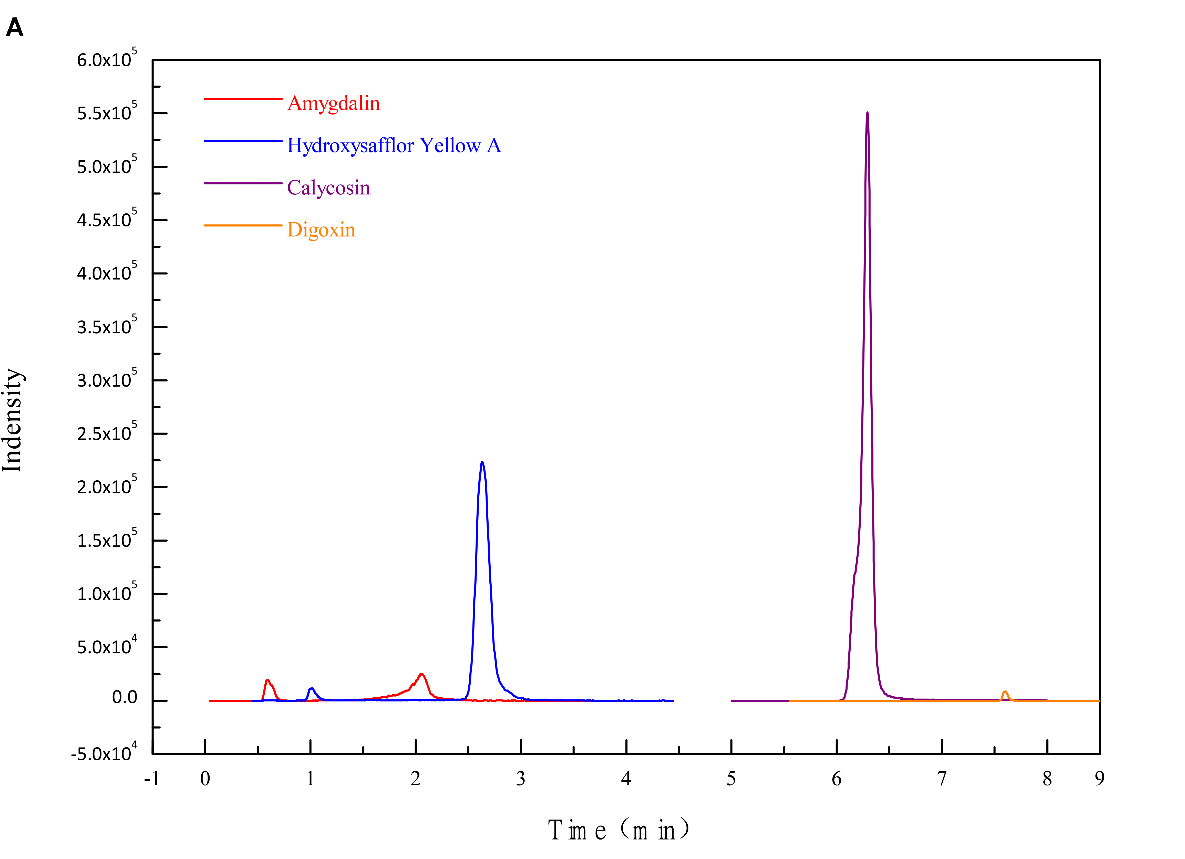
**

**
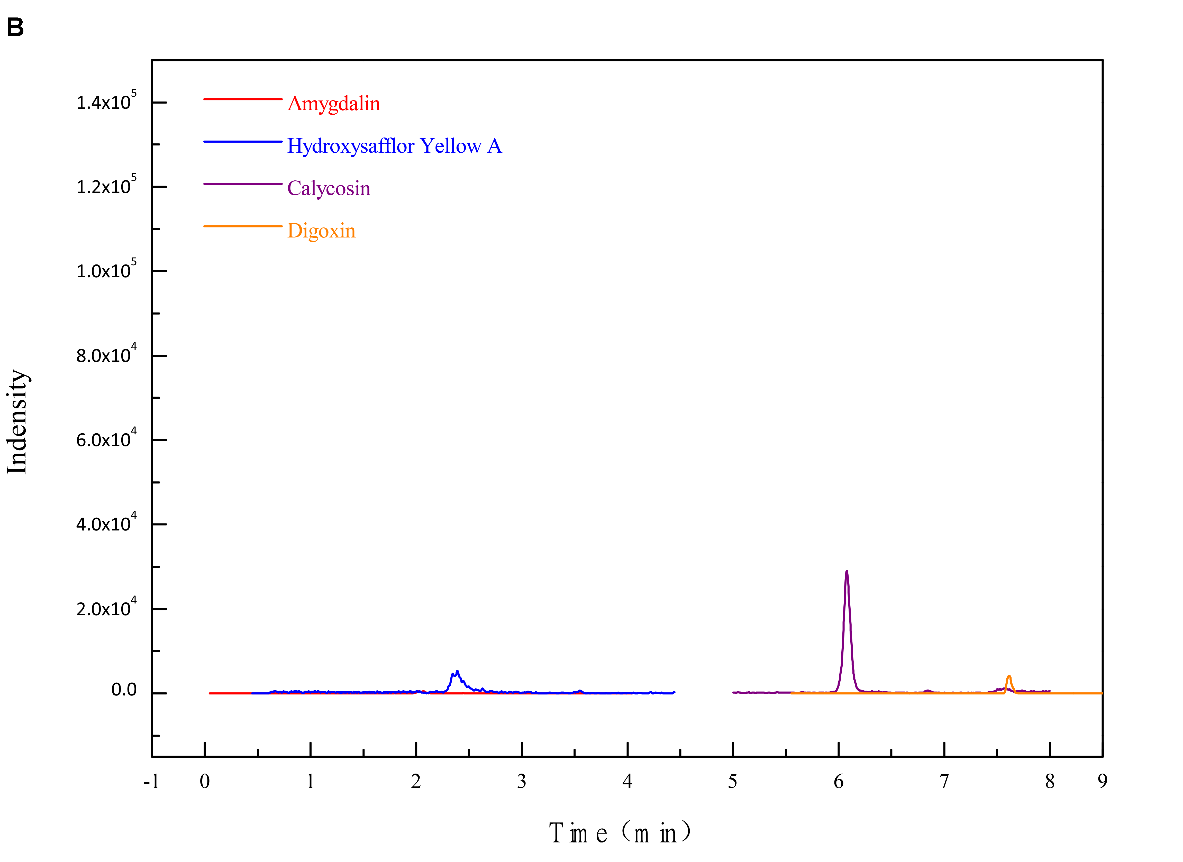
**

**Figure S1.** LCMS analysis of BYHWD components. LCMS chromatograms of three main components (Amygdalin, Hydroxysafflor Yellow A, Calycosin, and Digoxin) of blank plasma plus standard agents (A) and Sham+ BYHWD (B) in rat’s plasma sample.

###
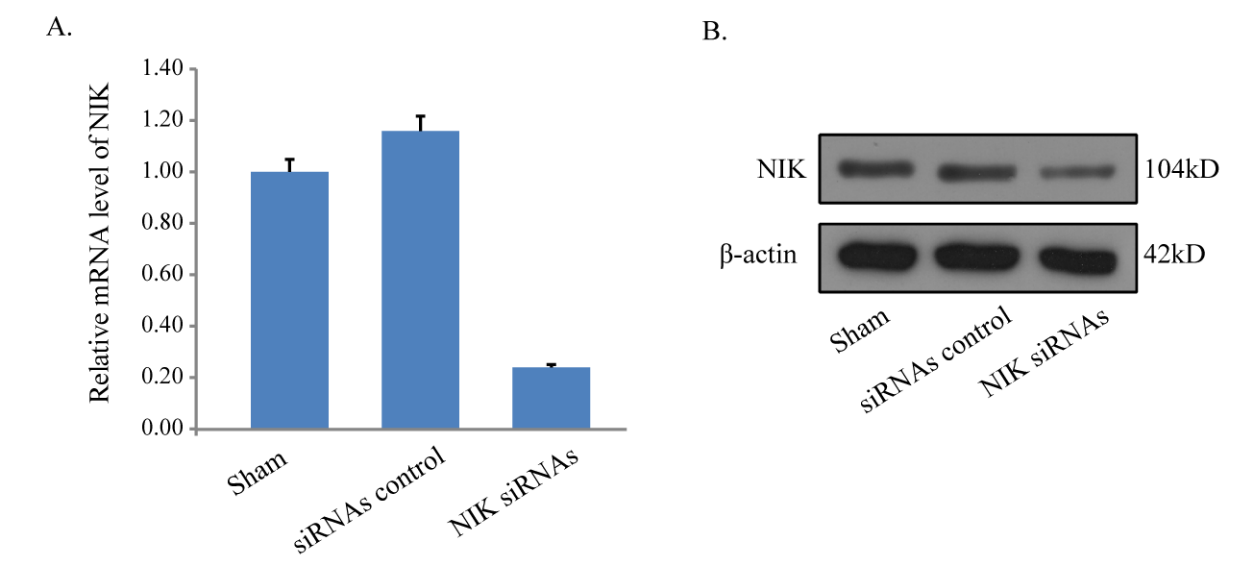


**Figure S2.** Effect of NIK siRNAs on NIK expression were assessed by pre-experiments. The mRNA expression of NIK detected by RT-qPCR and protein level of NIK by Western blot. Transfection efficiency of NIK siRNAs was over 40%.
